# Ovary does harbor stem cells - size of the cells matter!

**DOI:** 10.1186/s13048-020-00647-2

**Published:** 2020-04-17

**Authors:** Deepa Bhartiya, Diksha Sharma

**Affiliations:** Stem Cell Biology Department, ICMR-National Institute for Research in Reproductive Health, Jehangir Merwanji Street, Parel, Mumbai, 400 012 India

**Keywords:** Ovary, Stem cells, Very small embryonic-like stem cells (VSELs), Ovarian stem cells (OSCs)

## Abstract

A recent study published in the journal *Nature Communications* from Karolinska Institute, Sweden was unable to detect stem cells in adult human ovarian cortex by single-cell RNAseq and by studying cell surface antigen profiles by flow cytometry studies. Their findings are startling since stem cells have been well characterized in the adult mammalian ovary of several species including mouse, rabbit, monkey, sheep, pig and humans. Ovarian stem cells include pluripotent, very small embryonic-like stem cells (VSELs) and slightly bigger ovarian stem cells (OSCs) which are easily visualized in smears obtained by gently scraping the ovary surface. The potential of ovarian stem cells to differentiate into oocyte-like structures in vitro and also resulting in the birth of mouse pups has been reported. A possible role of ovarian VSELs in initiation of ovarian cancers has also been delineated. The ovarian stem cells can also be collected by enzymatic digestion of ovarian tissue for various studies, taking care to always pellet the cells suspension at 1000 g since this high speed is required to collect the small-sized stem cell populations (VSELs & OSCs) with high nucleo-cytoplasmic ratio. These stem cells invariably get discarded when cells suspension is spun at lower speed. The cells were spun at 300 g for various experiments in the Karolinska study and this is the underlying reason for their negative results. Stem cells were inadvertently and unknowingly discarded and never got analyzed by single-cell RNAseq and flow cytometry experiments. To conclude, stem cells surely exist in adult mammalian ovary and their role during neo-oogenesis and primordial follicle assembly under physiological conditions is currently being investigated.

## Main text

**Fig. 1:**
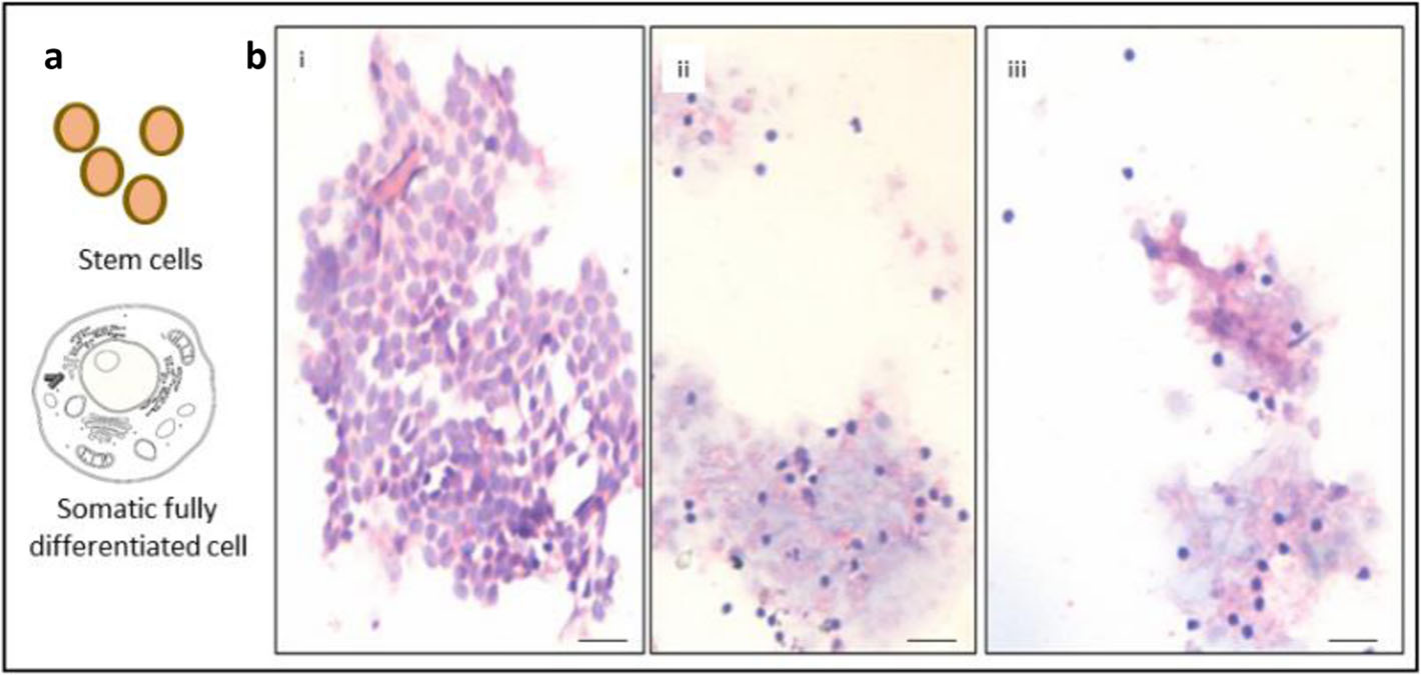
**a** Schematic representation of stem cells compared to fully differentiated somatic cell (not drawn to scale). Stem cells are quiescent, spherical cells of small size and with high nucleocytoplasmic ratio. When cells suspension is centrifuged, the somatic cells pellet down at 300 g (and care is taken not to spin at a higher speed in order to protect them from rupturing). However, at this speed, the stem cells remain buoyant and get unknowingly discarded. **b** Surface epithelial cells gently scraped from sheep ovary after Hematoxylin & Eosin staining. One can see sheets of epithelial cells and at places small, spherical, darkly stained putative stem cells. These stem cells have been characterized in details elsewhere [2, 3]

The controversy surrounding ovarian stem cells does not seem to end. Recently a group from Karolinska Institute, Sweden carried out single-cell RNA-seq and transcriptome analysis to study human ovarian cortical tissue and concluded that it does not harbor stem cells and rather comprises six distinct cell types including oocytes, granulosa cells, immune cells, endothelial cells, perivascular cells, and stromal cells [1]. On the contrary, ovary harbors two populations of stem cells including pluripotent, 2–6 μm, very small embryonic-like stem cells (VSELs) and 6–8 μm ovarian stem cells (OSCs) [2]. These stem cells can easily be enriched by scraping the ovary surface in case of sheep (Fig. 1), rabbits, marmosets as well as in women whereas in mice (since the ovary is small in size) stem cells can be studied after enzymatic digestion [3–5]. Enzymatic digestion is in fact used to obtain single cells suspension of ovarian tissue of any species to study stem cells using specific markers by employing techniques like flow cytometry, RT-PCR etc.

Presence of smaller-sized cells (supposedly the VSELs) was recently confirmed in mice [6] and human [7, 8] ovaries by other groups as well and more than 20 groups have now independently reported VSELs in multiple adult tissues [9]. VSELs in mouse ovaries express pluripotent [OCT-4A, SOX-2, NANOG, SSEA-1 (SSEA-4 in humans)] and primordial germ cells [STELLA, FRAGILIS] specific markers whereas OSCs express cytoplasmic OCT-4B, SCA-1 (CD133 in humans), DDX-4 (MVH) and other markers. VSELs can be studied by flow cytometry as small sized cells with a surface phenotype of LIN-CD45-SCA-1+ in mice and LIN-CD45-CD133+ in human tissues [10]. Ovarian VSELs undergo rare asymmetrical cell divisions to self-renew and give rise to the OSCs which in turn undergo symmetrical cell divisions and clonal expansion to form germ cell nests before undergoing meiosis and differentiating into oocytes and this process is regulated by FSH [2, 11]. Ovarian stem cells have been reported to differentiate in vitro into oocytes [12, 13], birth of mouse pups [6] and their role in ovarian cancers has also been demonstrated [14]. Initial data is also published to support neo-oogenesis in adult ovaries in vivo from the stem cells [15–17].

DDX-4 is expressed on the OSCs/germ cells [6] and getting its expression on the perivascular cells by Wagner’s group [1] is suggestive of non-specific binding and technical issues. A careful study of the methodology by Wagner’s group [1] makes it very evident that the ovarian stem cells were unknowingly discarded while processing samples for single-cell RNA-seq and this could explain their negative results. We need to understand that stem cells differ from differentiated cells in size (Fig. 1) and metabolic state since the stem cells remain relatively quiescent and metabolically inactive. Differentiated cells, on the other hand, have abundant cytoplasm full of organelles crucial for their normal functions and metabolic status. The stem cells are much smaller in size, spherical in shape with minimal cytoplasm, scanty organelles and high nucleo-cytoplasmic ratio. This was first noticed by ultra-structural studies on the stem/ progenitor cells [9, 10]. Being relatively quiescent, stem cells do not require large number of mitochondria and other organelles. We have shown that the stem cells including both VSELs and OSCs pellet down only when spun at 1000 g rather than 300 g [3–5]. This higher speed does not affect the stem cells as they have minimal cytoplasm. This is the basic issue with study reported by Wagner et al.^1^ where they did all processing at 300 g as mentioned in their Material and Methods section. Like Wagner’s group, even Tilly’s group uses 300 g to study OSCs but suggest to increase speed to 600 g if number of DDX-4 positive cells is not enough [18]. Use of 600 g may lead to pelleting down of OSCs but VSELs will remain elusive even when cells are spun at 600 g and require a higher speed of 1000 g to pellet down. Stem cells possibly never got collected and analyzed by RNA-seq and flow cytometry studies leading to an erroneous conclusion that ovary does not harbor stem cells [1].

This use of a low speed to process stem cells has led to several controversies in the published literature. A review [19] published in Nature and also a research study [20] in Journal of Clinical Investigation concluded that there are no stem cells in adult pancreas whereas our group recently reported [21] that both the pancreas and also an enriched population of pancreatic islets harbor stem/progenitor cells including VSELs and PSCs (pancreatic stem cells) taking care to always spin cells at 1000 g while processing. We also observed that the numbers of endogenous stem cells increase in a diabetic, streptozotocin treated mouse pancreas in an attempt to restore homeostasis, but are unable to do so and hence the disease. This result suggests that rather than the stem cells, it is their niche that gets compromised under diabetic conditions and has led to evolving alternative strategies to treat diabetes [21].

It is because of this reason (unknowingly discarding VSELs during processing) that CD34+ cells isolated from bone marrow or cryopreserved from cord blood are only able to restore hematopoiesis and treat blood diseases and show no regenerative potential to transdifferentiate into non-hematopoietic cell types. HSCs are lineage-restricted and tissue-committed progenitors whereas the pluripotent VSELs with the true regenerative potential invariably get discarded along with the red blood cells after density gradient centrifugation [22] or during processing when cells are spun at 300 g. The controversy that erupted in 2006 regarding VSELs [23] and in 2018 regarding cardiac stem cells [24] was conceivably because of this basic reason. There is no doubt that pluripotent VSELs exist in multiple adult tissues including ovaries, pancreas and cardiac tissue. This technical glitch that stem cells require a higher speed of 1000 g to pellet down whereas somatic cells pellet down at 300-350 g needs to be settled and will result in a paradigm shift in the field of regenerative medicine.

We tried publishing our explanation for the negative results published by Wagner’s group in Nature Communications, but did not succeed. There is no need to use sophisticated techniques to demonstrate presence of stem cells in the adult ovaries when one can show their presence by simple scraping the ovarian surface (Fig. 1).

## Data Availability

Not applicable.
